# Multimodal-Imaging-Based Interpretable Deep Learning Framework for Distinguishing Brucella from Tuberculosis Spondylitis: A Dual-Center Study

**DOI:** 10.3390/diagnostics15232963

**Published:** 2025-11-22

**Authors:** Mayidili Nijiati, Mei Zhang, Chencui Huang, Xinyue Chou, Lingyan Shen, Haiting Ma, Zhenwei Ren, Maimaitishawutiaji Maimaiti, Yi You, Xiaoguang Zou, Yunling Wang

**Affiliations:** 1 Department of Radiology, The First Affiliated Hospital of Xinjiang Medical University, Urumqi 830002, China; mydl0911@163.com; 2 Department of Radiology, Xinjiang Medical University Affiliated Fourth Hospital, Urumqi 830002, China; zmxjzyy@163.com (M.Z.); 15699120718@163.com (X.C.); mht881022@163.com (H.M.); 13109910153@163.com (Z.R.); 3 Xinjiang Key Laboratory of Artificial Intelligence Assisted Imaging Diagnosis, Kashi 844000, China; zxgks@163.com; 4 Department of Deepwise AI Lab, Hangzhou Deepwise & League of PHD Technology Co., Ltd., Hangzhou 830054, China; huangchencui@deepwise.com (C.H.); shenlingyan@deepwise.com (L.S.); youyi@deepwise.com (Y.Y.); 5 Department of Spinal Surgery, The First People’s Hospital of Kashi Prefecture, Kashi 844000, China; sawut0998@163.com; 6 Department of Pharmacy, The First People’s Hospital of Kashi Prefecture, Kashi 844000, China

**Keywords:** tuberculosis spondylitis, brucella spondylitis, deep learning, multimodal imaging

## Abstract

**Objectives:** Brucella spondylitis (BS) and tuberculosis spondylitis (TS) are two causes of infection that share overlapping clinical and imaging features, complicating diagnoses. Early differentiation is critical, as treatment regimens differ significantly. This study aims to develop a deep learning framework using multimodal computed tomography (CT) and magnetic resonance imaging (MRI) data to accurately distinguish between these two conditions, improving diagnostic accuracy and patient outcomes. **Methods:** In this study, imaging data were acquired from two centers using different MRI and CT protocols. Sagittal T1-weighted (T1WI) and T2-weighted imaging (T2WI), fat-suppression sequences (T2WI FSE), and sagittal CT data were collected. Image preprocessing included region of interest (ROI) segmentation, and normalization and augmentation techniques were used. A deep learning model, based on pre-trained GoogleNet architectures, was trained and evaluated against human radiologists using metrics including accuracy, sensitivity, and AUC to assess diagnostic performance. **Results:** In this study, the GoogleNet deep learning model outperformed other architectures in classifying TS and BS, achieving AUCs of 95.97%, 91.24%, and 81.25% across training, test, and external validation datasets, respectively. In contrast, ResNet, DenseNet, and EfficientNet models showed lower AUC values. GoogleNet also demonstrated high accuracy (90.77% training, 83.04% test) and 90.91% sensitivity and 61.11% specificity in external validation. When compared to three radiologists, GoogleNet outperformed in diagnostic accuracy and speed, achieving an AUC of 88.01% and processing cases in 0.001 min. These findings highlight the potential of AI to enhance diagnostic performance and efficiency. Lastly, the explanation provided by the Grad-Cam model precisely localized major lesions. **Conclusions:** This multimodal-imaging-based deep learning model could well differentiate TS and BS. Deep learning does not need manual feature extraction, selection, or model development, and has great potential in daily clinical practice.

## 1. Introduction

Infectious spondylitis, an inflammatory condition of the spine, can lead to severe outcomes such as chronic pain, spinal deformity, and neurological impairment if not diagnosed and treated promptly [1]. Among the various causes of spondylitis, brucella spondylitis (BS) and tuberculosis spondylitis (TS) spondylitis are two major infectious etiologies, each posing unique diagnostic challenges. Both conditions predominantly affect the vertebral bodies and can exhibit overlapping clinical and imaging features, making early differentiation essential for effective treatment and prevention of long-term complications [2,3].

BS and TS are particularly prevalent in regions with high infection rates, BS are prevalent in areas with extensive livestock farming [4], while TS is common in limited healthcare areas. Both diseases are endemic in parts of the developing world, where they pose significant public health challenges [5]. Patients often present with similar symptoms, including back pain, fever, and weight loss, along with complications like vertebral collapse, paravertebral abscesses, and spinal cord involvement [6]. However, because these infections share overlapping clinical manifestations and imaging findings, misdiagnosis is common, which can lead to delayed or inappropriate treatment and consequently worse patient outcomes [7,8]. This not only impacts individual health but also imposes significant social and economic burdens on healthcare systems due to prolonged illness and increased hospitalizations.

Differentiating BS from TS is critical, as the treatment regimens for these infections differ substantially [9]. Despite advances in diagnostic tools, distinguishing between these two conditions remains a clinical challenge. Conventional imaging techniques, such as computed tomography (CT) and magnetic resonance imaging (MRI), are essential in diagnosing spondylitis. CT provides detailed images of bone structures, helping to identify vertebral destruction and abscess formation, while MRI is highly sensitive to soft tissue changes and can detect early inflammatory processes in the spine [10]. However, both imaging modalities have limited ability to differentiate BS from TS due to the similarity of their imaging features, including vertebral collapse and the presence of paravertebral abscesses [11,12].

Recent advancements in deep learning, a branch of artificial intelligence, offer new possibilities in medical imaging by enabling automated analysis of complex imaging patterns [13,14]. Deep learning models have already demonstrated promise in differentiating various infectious conditions, including spinal infections [15]. These models can process multimodal imaging data more effectively than traditional methods, potentially uncovering subtle features that might be missed by human clinicians.

This study seeks to develop a deep learning framework that leverages multimodal imaging data, CT and multiparametric MRI to distinguish BS from TS. By conducting a two-center study, we aim to enhance the robustness and generalizability of the framework, ensuring its applicability across diverse clinical settings and improving diagnostic accuracy and patient outcomes.

## 2. Materials and Methods

### 2.1. Consent to Participate

This study, conducted at The First People’s Hospital of Kashi Prefecture and Xinjiang Medical University Affiliated Fourth Hospital, received ethical approval from the institutional review board. Given the retrospective nature of the research, written informed consent from participants was deemed unnecessary. All data were handled in accordance with ethical standards, ensuring the confidentiality and anonymity of the participants.

### 2.2. Study Population

This study was approved by the Ethics Committee of the Fourth Hospital Affiliated with Xinjiang Medical University and the First People’s Hospital, which waived the requirement for written informed consent. We conducted a retrospective cohort study from January 2020 to December 2024, involving 195 patients diagnosed with either BS or TS at Xinjiang Medical University Affiliated Fourth Hospital, serving as the training set. An additional 43 patients from the First People’s Hospital formed the external validation cohort.

All participants underwent routine assessments, including blood cultures, tuberculin tests, T-spot TB tests, standard tube agglutination titer tests, and chest X-rays. Demographic and clinical data were extracted from the Electronic Medical Record System. Accurate diagnosis of BS required verification through clinical manifestations, imaging characteristics, and laboratory findings.

Inclusion criteria comprised: (1) presentation of relevant clinical symptoms for at least six months; (2) a positive agglutination test with a titer of 1:160 or higher, positive blood culture, or identification of brucella spp. from tissue samples or histopathological examination; (3) evidence of at least one morphological or signal abnormality on MRI; and (4) availability of complete medical records.

Exclusion criteria included: (1) relevant clinical symptoms for less than six months without prior treatment before MRI; (2) incomplete MRI acquisition; (3) lack of clinical or histopathological data; (4) suboptimal image quality due to motion or susceptibility artifacts; and (5) spondylitis due to alternative causes, such as pyogenic infections, malignant tumors, lumbar disc herniation, non-bacterial infections (e.g., fungal), non-infectious lesions, unidentified pathogens, or incomplete information regarding the infectious agent.

### 2.3. MR and CT Image Acquisition Scheme

The imaging parameters varied between two centers in the study. At Center 1, T1-weighted imaging (T1WI) was performed on a Siemens Skyra 3.0T with a repetition time (TR) of 600 ms, echo time (TE) of 9.5 ms, a field of view (FOV) of 320 mm, and a matrix size of 256 × 256. The slice thickness and spacing were set to 4 mm and 1 mm, respectively. T2-weighted imaging (T2WI) utilized a TR of 3000 ms and a TE of 88 ms, maintaining the same FOV and matrix size as T1WI. Fat-suppressed T2-weighted imaging (T2WI FSE) had a TR of 3600 ms and a TE of 83 ms, with a reduced FOV of 300 mm while keeping the matrix size and slice parameters consistent.

In contrast, Center 2 employed a Siemens MAGNETOM Aera 1.5T, where T1WI was acquired with a TR of 520 ms and TE of 8.4 ms, using a FOV of 300 mm and similar matrix size and slice parameters as Center 1. T2WI mirrored the TR of 3000 ms and TE of 94 ms, while T2WI FSE was performed with a TR of 3700 ms and TE of 85 ms, again maintaining the same matrix size and slice thickness.

CT scans were conducted using a GE VCT 64-slice scanner (GE Healthcare, Chicago, IL, USA) and a Siemens SOMATOM scanner (Siemens Healthineers, Erlangen, Germany) for training and external testing sets, respectively. A tube voltage of 120 kV was applied, with the tube current automatically adjusted for optimal image quality. The rotation speed was set to 0.6 s, resulting in an average slice thickness of 2.5 mm for all helical mode CT scans. Detailed information is provided in Table 1.

### 2.4. Image Process

All patients underwent CT and MRI (T1WI, T2WI, T2WI FSE) examinations. ROI segmentation was performed by two experienced musculoskeletal radiologists (Mei Zhangand Xinyue Chou, each having more than 10 years and 15 years of experience) using the Deepwise Multimodal Research Platform version 2.6.4 (https://keyan.deepwise.com). When there was a significant disagreement between the two radiologists on the boundary issues, the two radiologists reached a consensus after discussion. To quantify the inter-annotator agreement, a representative subset of 20 cases was randomly selected from the dataset. For these cases, Dice coefficients were calculated between the independent ROI annotations of the two radiologists. Using a bootstrap method, the 95% confidence interval for the inter-annotator Dice agreement was estimated to be 0.73–0.84, indicating moderate to good consistency between the two radiologists.

After collecting and annotating the images, the original CT and MR (T1WI, T2WI, T2WI FSE) image files were downloaded and quality-checked, including checking the value range and confirming whether the image orientation was normal. Additionally, since the images came from multiple centers and different machines, the differences in pixel shape were also counted to determine whether resampling was needed to unify the data to the distribution of most samples. The CT images were adjusted according to winwidth = 1800 and wincenter = 400, and min–max normalization was used for calculation. MR images were not adjusted for window width and position, and min–max normalization was used directly. Given that clinical experts annotated the regions of interest (ROIs) in three dimensions, we extracted the 2D slice with the maximum lesion cross-sectional area for subsequent processing. To retain additional contextual imaging features surrounding the lesions, we then cropped a region encompassing each labeled lesion with an expansion factor of 1.3 relative to the lesion’s bounding box dimensions.

Before training, we first resized the images to 256 × 256 pixels to ensure that all input images had a consistent size. Then, we randomly flipped the images horizontally with a probability of 40% to increase data diversity. In addition, we randomly rotated the images with a rotation angle range of −60 to 60 degrees to further enhance data diversity. To adjust the color characteristics of the images, we applied color jittering to randomly change the brightness, contrast, saturation, and hue of the images, with specific parameters of brightness 0.2, contrast 0.2, saturation 0.2, and hue 0.1. After that, we randomly cropped a 224 × 224 pixel area from the adjusted images to simulate different perspectives and points of interest. Finally, we converted the images to tensor format and normalized them, using the mean [0.485, 0.456, 0.406] and standard deviation [0.229, 0.224, 0.225] to standardize each channel, to improve the stability and performance of model training.

After image enhancement, each sequence image slice was loaded in RGB mode, and the CT and T1WI, T2WI, T2WI FSE data were merged into a 224 × 224 × 3 × 4 matrix, which is called a 4-channel “stack”, to be further used for deep learning training programs. Specifically, each grayscale image in the sequence was replicated across the three channels to form a 3-channel tensor with identical values in all channels, and normalization was performed using consistent constants across all sequences.

### 2.5. Model Train

The dataset was derived from two different centers. We randomly divided the patients from Center 1 into 7:3 groups. This ensures that all slices from a single patient are entirely contained within either the training or test set; no patient’s slices are split between the two datasets, while data from Center 2 was used exclusively for external validation.

**Figure 1:**
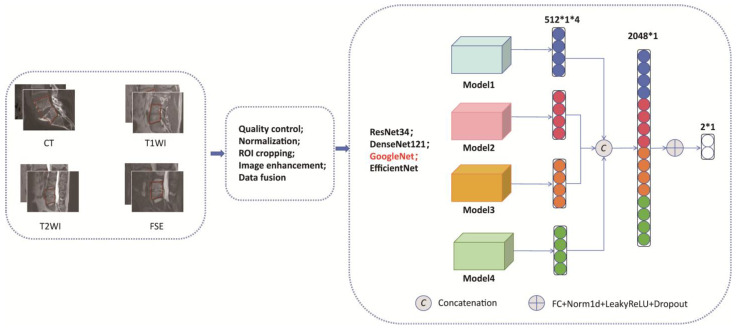
Multimodal deep learning framework for medical image analysis. The red frame is region of interest (ROI).

We utilized a pre-trained GoogleNet (ResNet34, DenseNet121, EfficientNet) as the feature extractor and independently extracted features of each sequence image in the stack. We constructed a module list containing multiple GoogleNet (ResNet34, DenseNet121, EfficientNet) feature extractors, which shared the same pre-trained weights but were fine-tuned independently during training. The final best model was selected based on a two-step criterion applied to the test set (not the external validation set, to avoid data leakage): (1) the test loss plateaued (relative change < 1% over 10 consecutive epochs), and (2) the test AUC stabilized within ±0.01. As shown in Figure 1, the features obtained from the feature extractors were concatenated along a single channel dimension, then classified by a fully connected classifier, which consisted of multiple fully connected layers, batch normalization layers, LeakyReLU activation functions, and Dropout layers, ultimately outputting the probability distribution corresponding to the classes. The Focal Loss function was used to calculate the loss between the model’s output and the true labels, with the equation defined as: FL(pt) = −αt(1 − pt)γlog(pt). This represents the predicted probability for the target class (with logits converted to probabilities via the sigmoid function). Here, the “positive class” refers to samples labeled as tuberculous spondylitis (TS), and the “negative class” refers to brucellar spondylitis (BS). The parameters were set as alpha = 0.5 (weight for positive class), gamma = 2 (focusing parameter), and reduction = ‘mean’ (to avoid numerical instability). This parameter selection was determined by comparing validation loss and precision-recall curve (PRC) area across alpha values (0.3, 0.4, 0.5) and gamma values (2, 3). The Adam optimizer was employed, with an initial learning rate set to 1 × 10^−3^ and weight_decay set to 0.0001. A StepLR learning rate scheduler dynamically adjusted the learning rate every 10 epochs (milestones at epochs 10, 20, 30, …) by multiplying the current rate by gamma = 0.1; no warm-up phase was used as preliminary experiments showed it did not improve convergence. During training, the model was trained on the training set with a batch size of 32, using a fixed random seed to ensure reproducibility. Training was conducted over 50 epochs, with 3 independent runs performed to account for stochasticity, and the optimal model (45th epoch) was selected based on balanced performance across training and test set metrics. Determinism settings were enabled via torch.backends.cudnn.deterministic = True to minimize random variation. Training was conducted using an A100 GPU (NVIDIA) on a Linux system (Ubuntu version 7.5.0) with CUDA version 11.4. Open-source software was used, including Python (version 3.7.7; Python Software Foundation), Pytorch version 1.4.0, and torchvision version 0.5.0.

Figure 1 shows the model development in this study.

Figure 1 presents a comprehensive pipeline for integrating multimodal medical imaging data using deep learning. (A): Input modalities (CT, T1WI, T2WI, T2WI FSE) undergo preprocessing steps such as quality control, normalization, ROI cropping, image enhancement, and data fusion. (B): Four parallel CNNs-ResNet34, DenseNet121, GoogleNet, and EfficientNet-are deployed to extract features from each modality. (C): Features from the CNNs are concatenated into a unified 2048-dimensional vector. (D): The concatenated features are processed through FC layers, normalization, LeakyReLU activation, and dropout regularization, yielding a binary classification output. The diagram emphasizes the framework’s modular design and end-to-end processing workflow, underscoring its potential for leveraging diverse imaging data to improve diagnostic accuracy.

### 2.6. Model Evaluation

The model’s performance was evaluated using six metrics: accuracy, precision, sensitivity, F1 score, specificity, and AUC. Accuracy is the proportion of correctly classified samples out of the total number of samples. Precision represents the ratio of correctly classified positive samples to the actual number of positive samples. Sensitivity indicates the ratio of correctly classified positive samples to the actual number of positive samples. Specificity represents the ratio of correctly classified negative samples to the actual number of negative samples. AUC is the area under the ROC curve, which is obtained by plotting the True Positive Rate (TPR) on the *Y*-axis and the False Positive Rate (FPR) on the *X*-axis. The range of AUC values is from 0.5 to 1, with higher AUC values indicating better classifier performance. To prevent overfitting, we implemented an early stopping strategy during training and monitored the model’s performance on the test set. Training continued until the loss on the test set no longer significantly decreased.

### 2.7. Human–Machine Comparison

To evaluate the model’s diagnostic performance compared to human experts, an independent test set was used to compare the optimal GoogleNet model with three radiologists having x, x, and x years of experience, respectively. The radiologists (Mayidili Nijiati and Haiting Ma, each having more than 20 years and 15 years of expertise in musculoskeletal radiologist) independently reviewed CT and MRI (T1WI, T2WI, T2WI FSE) images and provided their diagnoses based on clinical criteria. When there was a significant disagreement between the two radiologists on the boundary issues, the two radiologists reached a consensus after discussion. The AI model, trained on combined MR and CT sequences, was evaluated on the same test set.

The comparison focused on key diagnostic performance metrics, including accuracy, AUC, sensitivity, and specificity. Each radiologist reviewed the images independently without access to patient clinical information. The AI model’s predictions were generated automatically without manual intervention. Reading times for both radiologists and the AI model were recorded for efficiency analysis.

## 3. Results

### 3.1. General Information Analysis

In total, 238 patients were included in the study, and they had 261 TS lesions and 173 BS lesions; the age and sex demographic data are shown in Table 2.

### 3.2. Model Performance Comparison

**Figure 2:**
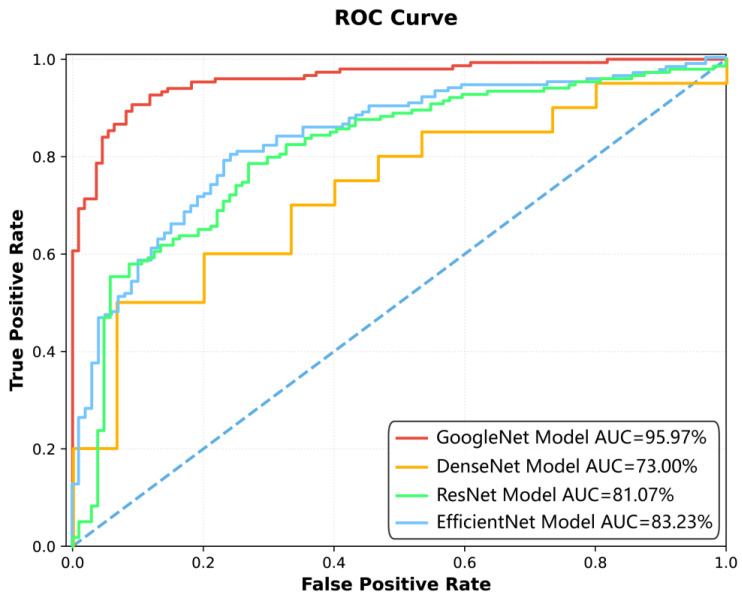
Comparison of model performance using ROC curves in the training set.

**Figure 3:**
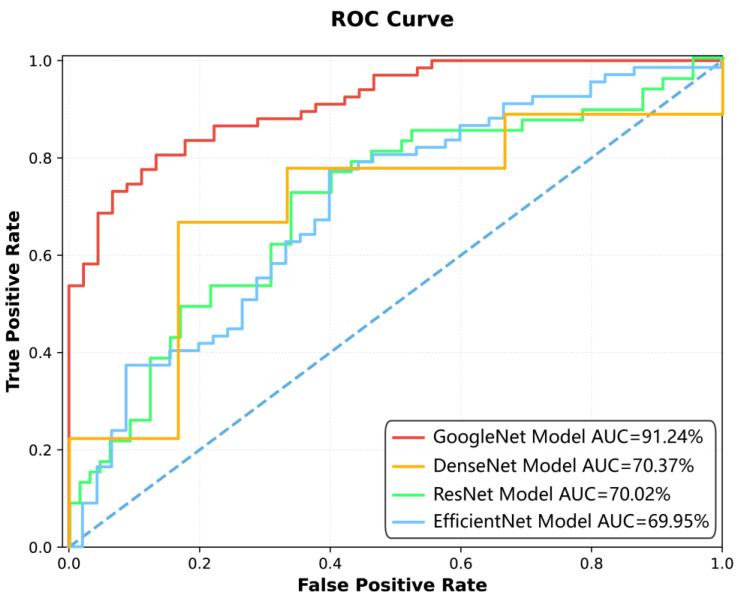
Comparison of model performance using ROC curves in the test set.

**Figure 4:**
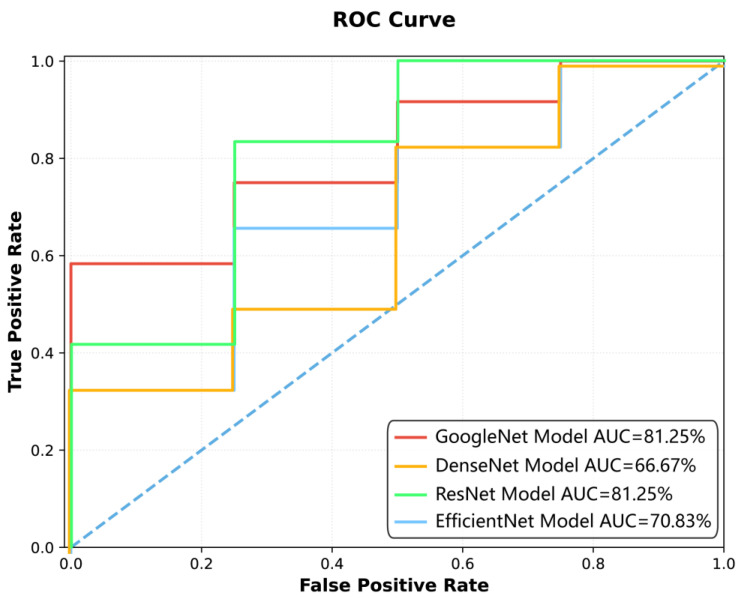
Comparison of model performance using ROC curves in the external test set (Blue dashed line represents random classifier performance (AUC = 0.5) serving as baseline for model comparison).

Among the four deep learning models constructed for spinal tuberculosis classification, the GoogleNet model demonstrated the highest diagnostic performance (Figure 2, Figure 3 and Figure 4, Table 3 and Table 4). It achieved AUCs of 95.97% [95.93%, 96.20%] on the training set, 91.24% [91.04%, 91.93%] on the test set, and 81.25% [76.04%, 87.94%] on the external validation dataset, indicating strong generalizability.

Figure 2: The ROC curve illustrates the performance of four different models—GoogleNet, DenseNet, ResNet, and EfficientNet—in terms of their ability to distinguish between positive and negative classes. The *x*-axis represents the False Positive Rate, while the *y*-axis represents the True Positive Rate. The area under the curve (AUC) is used as a measure of overall model performance, with higher values indicating better discrimination. The GoogleNet Model achieved the highest AUC of 95.97%, followed by EfficientNet (83.23%), ResNet (81.07%), and DenseNet (73.00%). The curves show that GoogleNet consistently outperformed the other models across various thresholds, as evidenced by its closer proximity to the top-left corner of the plot, which represents ideal performance. The visual comparison highlights the superiority of GoogleNet in minimizing false positives while maximizing true positives, making it the most effective model among those tested.

Figure 3 The ROC curve in Figure 3 illustrates the classification performance of four deep learning models—GoogleNet, DenseNet, ResNet, and EfficientNet—across varying decision thresholds. The *x*-axis denotes the FPR, while the *y*-axis represents the TPR. Each model’s performance is quantified by its AUC, with higher values indicating superior discriminative ability. The curves are plotted against a diagonal dashed line, which signifies random guessing (AUC = 50%). The visual comparison highlights the relative efficacy of the models in balancing TPR and FPR, with GoogleNet exhibiting the highest AUC (91.24%), followed by EfficientNet (69.95%), ResNet (70.02%), and DenseNet (70.37%). The proximity of each curve to the top-left corner of the plot reflects its ability to maximize true positives while minimizing false positives.

**Figure 5:**
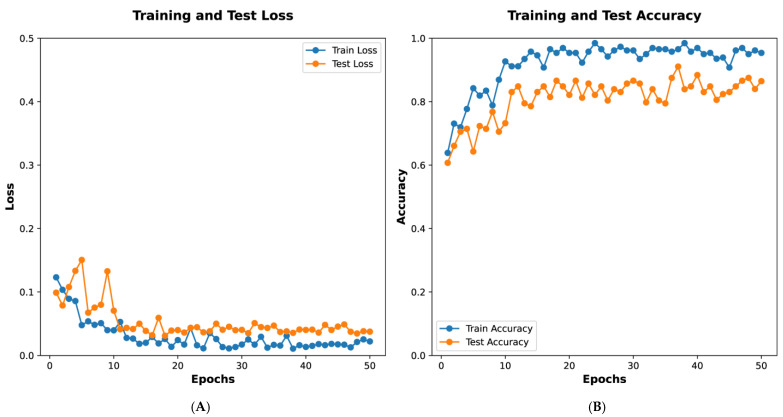
Training dynamics of the deep learning model. (**A**) Training and test loss; (**B**) Training and test accuracy.

Figure 5 depicts an ROC curve illustrating the performance of four deep learning models. The *x*-axis represents the False Positive Rate (FPR), while the *y*-axis denotes the True Positive Rate (TPR). Four distinct curves are plotted: GoogleNet (red line) and ResNet (green line) demonstrate the highest AUC values of 81.25%, indicating strong classification ability. DenseNet (orange line) shows a lower AUC of 66.67%, and EfficientNet (blue line) achieves an AUC of 70.83%. The dashed diagonal line serves as a baseline for random guessing. The curves highlight how each model balances TPR and FPR across different thresholds, with GoogleNet and ResNet consistently outperforming the others. The visual comparison provides insights into the models’ suitability for the specific classification task under study.

The ResNet model obtained AUCs of 81.07%, 70.02%, and 81.25% on the training, test, and external validation datasets, respectively. The DenseNet model showed AUCs of 73.00%, 70.37%, and 66.67%, while the EfficientNet model achieved AUCs of 83.23%, 69.95%, and 70.83% across the three datasets.

In terms of accuracy, the GoogleNet model reached 90.77% [89.77%, 91.77%] on the training set, 83.04% [82.97%, 83.87%] on the test set, and 81.25% [76.67%, 83.33%] on external validation. The ResNet model achieved 76.45%, 68.75%, and 87.50%, while the DenseNet and EfficientNet models had test set accuracies of 66.67% and 66.96%, respectively.

On the external validation dataset, the GoogleNet model achieved 61.11% specificity (outperforming ResNet and EfficientNet) and maintained 90.91% sensitivity, indicating meaningful diagnostic performance despite the small sample size of 43 patients.

Figure 5A depicts training and test loss over 50 epochs. The *x*-axis represents the number of epochs, while the *y*-axis shows the loss values. The blue line indicates the training loss, which decreases steadily, demonstrating the model’s progressive learning. The orange line represents the test loss, which also declines with minor fluctuations, indicating the model’s ability to generalize to unseen data. Figure 5B illustrates training and test accuracy over the same period. The blue line shows training accuracy, which rises sharply and stabilizes near 1.0, reflecting strong performance on the training dataset. The orange line depicts the test accuracy, which increases and stabilizes at a high level, albeit with slight variability, suggesting effective generalization. These graphs collectively demonstrate the model’s effective training and validation performance.

Figure 5 presents the training process of the GoogleNet model, showing that the loss function plateaued at the 15th epoch. The final model, selected at the 45th epoch based on accuracy and AUC, was subsequently applied for classification prediction in external datasets. Figure 3 and Figure 4 illustrate the classification performance of all models in testing and external validation set, emphasizing the superior generalization ability of GoogleNet.

**Figure 6:**
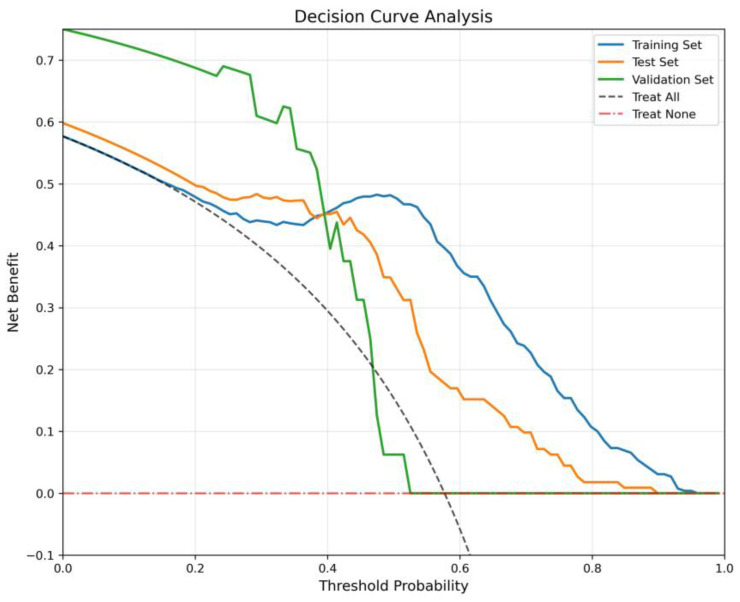
Decision Curve Analysis showing clinical net benefit across threshold probabilities. The Training Set and Test Set curves consistently demonstrate positive net benefit over a broad threshold range (0.0–0.8), peaking near 0.5, and outperform both “Treat All” and “Treat None” strategies. The Validation Set also maintains net benefit above these benchmarks despite minor mid-threshold fluctuations, confirming the model’s robust and clinically relevant decision-making performance across internal and external cohorts.

The Decision Curve Analysis (Figure 6) evaluates clinical net benefit across threshold probabilities, benchmarked against “Treat All” and “Treat None”. The Training Set and Test Set consistently yield positive net benefit across a wide threshold range (0.0–0.8), with peaks around 0.5, confirming robust decision-making utility. The Validation Set, despite minor fluctuations at mid-thresholds, maintains net benefit above the benchmarks, indicating clinical validity even in an external cohort.

### 3.3. Comparison of Diagnostic Performance Between Doctors and Artificial Intelligence

An independent test set, comprising 50 CT scans and MRI images (including T1WI, T2WI, and T2WI FSE sequences), was employed to compare the diagnostic performance of the optimal GoogleNet model with that of three radiologists who had advanced, moderate, and junior experience levels, respectively. As shown in Table 5, the senior, mid-level, and junior radiologists achieved AUCs of 77.5.00%, 72.00%, and 68.00% for TS diagnosis based on MR data and CT data. In contrast, the GoogleNet model, which utilized both MR and CT data, achieved an AUC of 88.01%, outperforming all radiologists.

In terms of accuracy, the advanced, moderate, and junior radiologists attained 68%, 64% and 62% for diagnosis The GoogleNet model demonstrated a superior accuracy of 72.00%. Sensitivity varied among the radiologists, with the junior radiologist achieving the highest sensitivity of 70%, though at the cost of lower specificity. The AI model exhibited a specificity of 95.24%, exceeding all human experts.

Additionally, the film-reading speed of the GoogleNet model was significantly faster than that of human radiologists, with an average processing time of 0.001 min per case, whereas the advanced, moderate, and junior radiologists required 5–11 min per case. These results indicate that AI-assisted diagnosis not only improves diagnostic accuracy but also significantly enhances efficiency compared to human experts.

### 3.4. Grad-Cam Model Explanation

**Figure 7:**
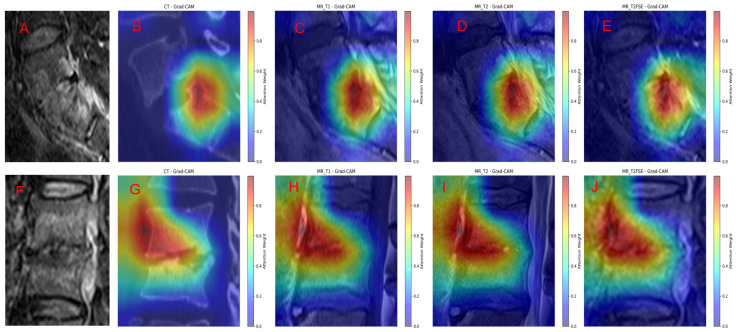
Grad-CAM Visualization of Model Attention in TS and BS. Grad-CAM overlays alongside original FS T2WI scans for representative cases of TS (**A**–**E**) and BS (**F**–**J**). In TS cases, model attention maps highlight vertebral lesions and extravertebral abscesses across CT and multiple MRI sequences, demonstrating close spatial alignment with pathological regions. For BS cases, heatmaps show focused activation on the vertebral lesions, confirming the model’s accurate localization of disease-specific abnormalities.

To enhance the interpretability of our model’s predictions, we applied Gradient-weighted Class Activation Mapping (Grad-CAM) to visualize the regions within different imaging modalities that most strongly influenced the classification decisions. Figure 7 illustrates representative cases of TS and BS alongside their respective Grad-CAM overlays across multiple sequences: FS-T2WI original scans (Figure 7A,F), as well as Grad-CAM heatmaps on T1WI, T2WI, FS T2WI, and sagittal CT images (Figure 7B–E for TS and Figure 7G–J for BS).

In TS cases (Figure 7A–E), the Grad-CAM visualizations reveal that the model’s attention is highly focused on the lesion site, including both vertebral involvement and associated extravertebral abscesses. The heatmaps demonstrate a strong spatial correspondence between areas of high model activation and radiologically apparent pathological findings, underscoring the model’s ability to localize and discern characteristic disease features. This consistency across various imaging modalities indicates robust lesion recognition by the model.

Similarly, in BS cases (Figure 7F–J), the Grad-CAM overlays reveal concentrated attention weights on the primary vertebral lesions, with prominent activation on sagittal CT and MR sequences. The model successfully highlights the core pathological regions, emphasizing lesion boundaries and tissue abnormalities consistent with BS. The comparably intense and localized activation patterns across all sequences further validate the model’s sensitivity to disease-specific imaging characteristics.

Overall, the Grad-CAM analysis confirms that GoogleNet effectively integrates multimodal imaging information to recognize and localize key pathological features in spinal infections. The alignment between Grad-CAM heatmaps and radiological abnormalities supports the model’s interpretability and provides visual evidence of its decision-making process. This interpretability is critical for clinical translation, as it enhances trust and facilitates diagnostic verification by correlating model focus areas with known disease manifestations.

## 4. Discussion

The study presented a comprehensive evaluation of a multimodal imaging deep learning framework, specifically the GoogleNet model, in differentiating between BS and TS. The findings revealed that the GoogleNet model achieved remarkable diagnostic performance, with area under the curve (AUC) scores of 95.97% on the training set and 91.24% on the test set, demonstrating its robustness and generalizability across diverse datasets. In contrast, other models such as ResNet, DenseNet, and EfficientNet exhibited notably lower AUCs, highlighting the superior capability of GoogleNet in this diagnostic task. Furthermore, the model’s specificity of 61.11% and sensitivity of 90.91% on external validation datasets reflect its diagnostic performance, with a notable emphasis on sensitivity. These results are pivotal, as they not only reinforce the efficacy of deep learning frameworks in medical imaging but also provide compelling evidence of their potential to enhance clinical decision-making in distinguishing complex conditions like spondylitis.

Additionally, the dual-center nature of the study contributes significantly to the robustness and generalizability of the results. By employing an independent test set in a multicenter context, the research underscores the potential for widespread application of the GoogleNet model across different clinical settings. The comparative analysis between the AI model and human radiologists further emphasizes the advantages of this technological approach; the GoogleNet model outperformed radiologists at varying levels of experience in terms of both accuracy and processing speed. While radiologists demonstrated AUCs ranging from 50% to 69%, the AI model achieved an AUC of 75%, showcasing its superior diagnostic capability. Moreover, the efficiency of the AI model, processing cases in mere seconds compared to the several minutes required by human experts, highlights the transformative potential of integrating AI into clinical workflows. Overall, the findings not only validate the effectiveness of the multimodal imaging deep learning framework but also suggest a paradigm shift in how diagnostic processes can be enhanced through advanced technology.

Differentiating BS from TS is a critical challenge in the fields of radiology and orthopedics due to the similar clinical and radiological presentations and different treatment approaches. The GoogleNet model, within a multimodal imaging deep learning framework, has demonstrated exceptional diagnostic performance in distinguishing BS from TS. It achieved AUC scores of 95.97% on the training set and 91.24% on the test set, with 86.67% specificity and 80.60% sensitivity, highlighting its potential to enhance clinical decision-making in complex diagnoses.

MRI has been utilized to identify distinct imaging characteristics between BS and TS. BS typically present with less damage and edge destruction in the lumbar spine, whereas tuberculosis is associated with severe vertebral body destruction, kyphosis, and multisegmented paravertebral abscesses. These differences are crucial for achieving accurate diagnosis and differentiation [16]. Deep learning models, such as 3D-ResNet and convolutional neural networks (CNNs), have been effectively used to distinguish tuberculosis from other conditions using imaging data. These models have shown high diagnostic accuracy, often surpassing human radiologists in identifying abnormalities in CT and chest X-ray images [17]. The integration of deep learning with image enhancement techniques further improves the detection and classification of tuberculosis, achieving high AUC scores and accuracy [18].

Conventional MRI can help differentiate BS from TS by identifying specific features. BS typically shows less severe vertebral destruction, vertebral posterior convex deformity, dead bone, and abscess scope compared to TS. Conversely, vertebral hyperplasia is more pronounced in BS than in TS [19].

Radiomics, which involves extracting a large number of features from medical images, can be used to differentiate BS from TS. Machine learning models using radiomics labels, such as random forest and support vector machine, have shown high accuracy in distinguishing between these conditions. These models outperform traditional MRI label-based logistic regression models, indicating the potential of radiomics in enhancing diagnostic accuracy [8].

The proposed multimodal-imaging-based deep learning framework has significant potential to enhance clinical decision-making in the diagnosis of spinal infections, particularly in distinguishing BS from TS. Misdiagnosis between these two conditions is a common challenge due to overlapping clinical presentations and imaging features, often leading to delayed or inappropriate treatment. By integrating multimodal imaging data, this framework leverages complementary information to improve diagnostic accuracy, thereby streamlining workflows for radiologists and clinicians. Early and accurate differentiation can facilitate timely administration of targeted therapies, ultimately improve patient outcomes and reduce the risk of complications associated with delayed or incorrect treatments.

Furthermore, this approach holds substantial promise in resource-limited settings, where BS and TS often coexist and access to advanced diagnostic tools may be restricted. The automated nature of deep learning models can alleviate the burden on healthcare professionals by providing consistent and reliable diagnostic support, even in areas with limited radiological expertise. Additionally, the cost-effectiveness of deploying such AI-driven tools could make them accessible in endemic regions, bridging the gap between technological advancements and on-the-ground clinical needs. By addressing these diagnostic challenges, the framework represents a transformative step toward precision medicine in infectious diseases, with the potential to significantly reduce the global burden of spinal infections. Furthermore, by integrating multimodal imaging and leveraging deep learning, this model can distinctly aid differentiation between spinal infections like BS and TS—especially critical where clinical overlap complicates diagnosis. Its automated, cost-effective support enhances accuracy and consistency, even in resource-limited settings, ultimately facilitating timely, targeted treatment and improving patient outcomes globally.

This study demonstrates several strengths and innovations, notably the integration of multimodal imaging data with deep learning, which represents a novel approach to distinguishing BS from TS. By combining diverse imaging modalities, the framework leverages complementary information, enhancing diagnostic accuracy beyond traditional methods. Additionally, the dual-center design is a key strength, as it improves the external validity of the findings by incorporating data from two independent institutions. This reduces potential biases associated with single-center studies and ensures the robustness and generalizability of the model. Together, these innovations mark a significant step forward in precision diagnostics for spinal infections. While satisfactory results were obtained, several limitations should be noted. First, the study was conducted at only two centers with a relatively small sample size; future research will involve a multicenter approach with a larger cohort. Second, important metrics such as laboratory and clinical data were not included; subsequent studies will incorporate multimodal data. Finally, other spinal infections, including pyogenic and fungal spondylitis, were not addressed; future investigations will expand to include these conditions. Third, ablation experiments to quantify the impact of image augmentation parameters (e.g., rotation range, intensity jitter) on model performance were not performed in the current work; future investigations will supplement such experiments to optimize augmentation strategies. Finally, other spinal infections, including pyogenic and fungal spondylitis, were not addressed; future studies will expand to include these conditions.

## 5. Conclusions

In conclusion, our research demonstrates that multimodal-imaging-based radiomics have significant potential for the differential diagnosis of BS and TS. The deep learning model enhances clinicians’ reliability in making accurate diagnoses, ultimately improving patient management and outcomes. Unlike traditional radiomics, which require manual feature extraction, selection, and classification, deep learning offers an end-to-end learning approach that is well-suited for daily clinical practice.

## Figures and Tables

**Table 1:** Scanner parameters in two settings.

Center	MR Scaner	Field Strength (T)	T1WI TR (ms)	T1WI TE (ms)	T2WI TR (ms)	T2WI TE (ms)	T2WI FSE TR (ms)	T2WI FSE TE (ms)	CT Scanner Model	CT Settings
1	Siemens Skyra (Siemens Healthcare, Engerland, Germany)	3.0	600	9.5	3000	88	3600	83	GE VCT 64-slice scanner (General Electric, Fairfield, CT, USA)	120 kV, auto mA, 0.6 s rotation, 2.5 mm slice thickness
2	Siemens MAGNETOM Aera	1.5	520	8.4	3000	94	3700	85	Siemens SOMATOM scanner	120 kV, auto mA, 0.6 s rotation, 2.5 mm slice thickness

**Table 2:** Baseline characteristics of the patients.

Group	Age	Sex (M)	Sex (F)
TS	49.58 ± 10.93	94 (61.84%)	58 (38.16%)
BS	56.07 ± 8.63	51 (59.30%)	35 (40.70%)

**Table 3:** Summary of Different Model performance comparison.

		AUC	Accuracy	Precision	Sensitivity	F1 Score	Specificity
GoogleNet Model	Train	95.97% [95.93%, 96.20%]	90.77% [89.77%, 91.77%]	93.15% [92.92%, 94.59%]	90.67% [89.92%, 94.59%]	91.89% [90.60%, 93.96%]	90.91% [89.74%, 92.66%]
	Test	91.24% [91.04%, 91.93%]	83.04% [82.97%, 83.87%]	90.00% [87.37%, 91.44%]	80.60% [76.06%, 87.58%]	85.04% [80.04%, 89.04%]	86.67% [77.73%, 87.78%]
	External Validation	81.25% [76.04%, 87.94%]	81.25% [76.67%, 83.33%]	85.11% [79.62%, 89.62%]	90.91% [90.12%, 92.09%]	87.91% [85.77%, 89.67%]	61.11% [41.74%, 62.66%]
ResNet Model	Train	81.07% [79.90%, 81.59%]	76.45% [74.45%, 78.45%]	81.33% [78.54%, 84.18%]	78.71% [75.71%, 78.71%]	80.00% [77.00%, 83.00%]	76.77% [74.76%, 78.73%]
	Test	70.02% [69.36%, 71.29%]	68.75% [0.5405, 0.7495]	67.65% [63.25%, 68.35%]	48.94% [43.48%, 54.07%]	56.79% [51.79%, 61.79%]	83.08% [75.00%, 86.56%]
	External Validation	81.25% [77.25%, 85.25%]	87.50% [79.00%, 89.67%]	85.71% [75.45%, 89.55%]	100.00% [66.67%, 100.00%]	92.31% [86.25%, 93.59%]	50.00% [37.50%, 87.50%]
DenseNet Model	Train	73.00% [71.43%, 76.79%]	65.71% [64.71%, 67.65%]	68.18% [66.67%, 71.43%]	75.00% [73.68%, 78.95%]	71.43% [70.00%, 73.17%]	53.33% [50.00%, 57.14%]
	Test	70.37% [68.37%, 72.37%]	66.67% [64.29%, 71.43%]	75.00% [71.43%, 80.71%]	66.67% [62.50%, 75.00%]	70.59% [66.67%, 75.00%]	66.67% [60.00%, 80.00%]
	External Validation	66.67% [57.64%, 75.69%]	50% [43.34%, 63.34%]	83.33% [80.00%, 87.75%]	41.67% [36.36%, 45.45%]	70.59% [50.66%, 73.56%]	75.00% [66.67%, 80.62%]
EfficientNet Model	Train	83.23% [80.08%, 83.70%]	80.00% [74.92%, 83.31%]	84.71% [83.62%, 85.26%]	82.61% [80.61%, 84.61%]	83.65% [81.65%, 85.65%]	75.76% [75.51%, 76.53%]
	Test	69.95% [69.30%, 71.05%]	66.96% [66.67%, 67.57%]	68.75% [68.35%, 69.62%]	82.09% [81.82%, 83.33%]	74.83% [74.48%, 75.34%]	44.45% [43.18%, 45.45%]
	External Validation	70.83% [63.19%, 80.21%]	75.00% [69.67%, 78.33%]	83.33% [79.00%, 84.33%]	83.33% [81.82%, 84.91%]	83.33% [78.33%, 88.33%]	50.00% [30.00%, 70.00%]

**Table 4:** The confusion matrix of the optimal model (level of lesion).

		True	TS	BS
Train	pred	TS	136	10
		BS	14	100
Test		TS	54	6
		BS	13	39
External Validation		TS	40	7
		BS	4	11

**Table 5:** Comparison of diagnostic performance between doctors and models.

Experience	Modality	AUC (%)	Accuracy (%)	Sensitivity (%)	Specificity (%)	Sample Number	Reading Time (min)
Advanced	MR + CT	77.5	68	63.33	75	50	5
Moderate	MR + CT	72	64	63.33	65	50	7
Junior	MR + CT	68	62	70	50	50	11
GoogleNet	MR + CT	88.01	72	55.17	95.24	50	0.001

## Data Availability

The original contributions presented in this study are included in the article. Further inquiries can be directed to the corresponding authors.
